# Supplementary material for: The Impact of Various Promotional Activities on Ebola Prevention Behaviors and Psychosocial Factors Predicting Ebola Prevention Behaviors in the Gambia Evaluation of Ebola Prevention Promotions
Source: Int J Environ Res Public Health. 2019 Jun 6;16(11):2020. doi: 10.3390/ijerph16112020 (PMC6603859; doi:10.3390/ijerph16112020)
Supplement: Supplementary file 1 [file ijerph-16-02020-s001.pdf]

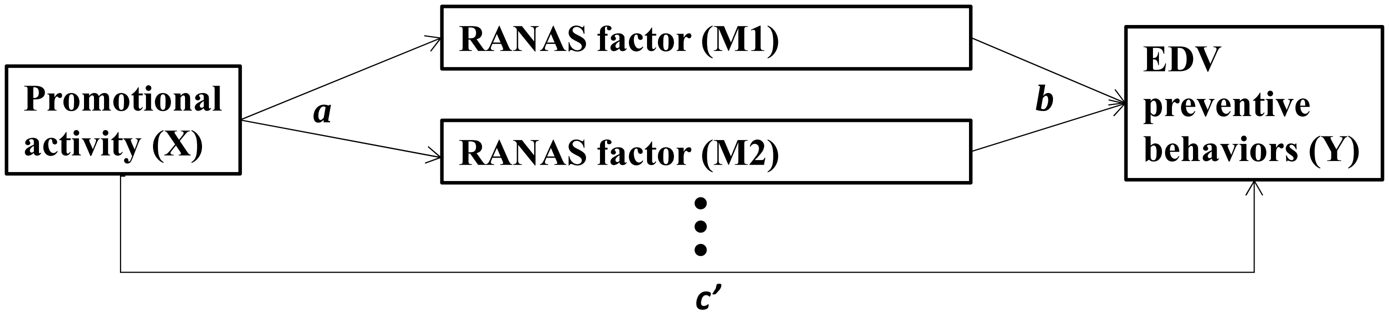

Figure S1. Template of a parallel multiple mediator model. RANAS psychosocial factors as mediators (M), promotion activities as predictors (X), and EDV preventive behaviors as outcomes (Y) are presented in mediator model. Calculations: (1) the direct effect (c`), the effect of the promotion activity on the EDV preventive behavior when the mediators are not present in the model (X on Y independent of M); (2) the specific indirect effect (a x b); (3) total effect (c), the sum of the specific indirect effect (a x b) and the direct effect (c`). Number of bootstrap samples for bias corrected bootstrap confidence intervals: 10'000. Level of confidence for all confidence intervals: 95% CL [LL, UL].
